# Efficacy and safety of perioperative application of esketamine on postoperative depression: a meta-analysis of randomized controlled studies

**DOI:** 10.1097/JS9.0000000000001870

**Published:** 2024-06-27

**Authors:** Yazhou Wen, Mingjie Mao, Ming Jiang, Qiaoqiao Liu, Qian Li, Xuan Wang, Hongmei Yuan, Xian Wang, Shanwu Feng

**Affiliations:** Department of Anesthesiology, Women’s Hospital of Nanjing Medical University, Nanjing Women and Children’s Healthcare Hospital, Nanjing, China

**Keywords:** postoperative depression, esketamine, meta-analysis

## Abstract

**Background:**

Postoperative depression has a profound impact on patients’ postoperative rehabilitation and overall quality of life. Preventing postoperative depression is of significant value because conventional antidepressants have a slow onset of action. Esketamine showed prompt and sustained antidepressant efficacy. Nevertheless, the safety and effectiveness of perioperative esketamine in preventing postoperative depression are still unknown. The purpose of this meta-analysis was to assess the safety and effectiveness of perioperative intravenous esketamine in relation to its ability to prevent postoperative depression.

**Materials and methods:**

Randomized controlled trials were searched in the following databases: Web of Science, Cochrane Central Registry of Controlled Trials, PubMed, and Embase. The primary outcome assessed is the postoperative depression scores. Postoperative pain ratings and adverse effects constituted secondary outcomes. Subgroup analyses were carried out on the basis of multiple variables, including the absence or presence of preoperative depression, the mode of esketamine administration, the dosage of esketamine, and the type of anesthesia.

**Results:**

A total of 16 studies encompassed 1161 patients who received esketamine intervention, whereas 1106 patients served as controls. Esketamine was efficacious in reducing postoperative depression scores when administered perioperatively, and the esketamine group maintained a lower postoperative depression score than the control group more than 4 weeks after surgery. Esketamine effectively alleviated postoperative pain scores without increasing the occurrence of postoperative nausea and vomiting, dizziness, drowsiness, nightmares, and dissociation.

**Conclusion:**

The administration of esketamine during the perioperative has the potential to decrease postoperative depression and pain scores without increasing the incidence of adverse effects.

## Introduction

Highlights

- Esketamine effectively reduced postoperative depression scores, and this effect persisted for an extended period.
- Esketamine effectively reduced postoperative pain scores.
- Esketamine did not increase the incidence of postoperative adverse effects (nausea and vomiting, dizziness, drowsiness, nightmares, and dissociation).

Postoperative depression is a common psychological issue that occurs in individuals after surgery. It is characterized by psychological stress reactions, including depression and anxiety^1^. The previous studies demonstrate that it has a significant impact on the effectiveness of postoperative rehabilitation and the general well-being of patients^2,3^. Postoperative depression is associated with postoperative pain, cognitive deterioration, extended inpatient length, and potentially reduced survival time^4–6^. More than 24% of surgical patients have reported experiencing perioperative depression^7^, with a proportion of nearly 47% in patients having cardiac surgery^8^. Unfortunately, there were few options for treating the emergence of depressive symptoms during the perioperative period or proactively preventing the occurrence of postoperative depression by administering antidepressants beforehand.

Ketamine is a traditional analgesic and sedative that is frequently used in anesthesiology for the induction and maintenance of anesthesia. Ketamine has demonstrated a prompt, sustained, and efficacious impact on treatment-resistant depression and major depressive disorder, according to a substantial body of evidence^9–13^. A recent meta-analysis demonstrated the efficacy of ketamine in reducing postoperative depression and pain scores while also revealing an increased occurrence of adverse effects^1^. Nevertheless, esketamine has progressively supplanted ketamine in clinical use, particularly following the approval of nasal esketamine by the United States Food and Drug Administration (FDA) as an additional treatment for major depressive disorder in 2019^14^. Furthermore, esketamine has been demonstrated in certain studies to have fewer adverse effects than ketamine^15–17^.

Esketamine, a racemic compound derived from ketamine, exerts notable and expeditious antidepressant properties^18–21^. Esketamine has nearly similar pharmacological characteristics as ketamine but with fewer side effects^15^. In a recent meta-analysis, our team discovered that esketamine effectively decreased the prevalence of postpartum depression (PPD) in women who had cesarean sections without raising the risk of side effects^22^. Despite the growing body of research in recent years concerning the perioperative use of esketamine^23–25^, its safety and effectiveness in treating postoperative depression in surgical patients remain unknown. Therefore, we conducted a meta-analysis on the perioperative use of esketamine for the prevention or treatment of postoperative depression and its associated adverse effects to provide a clinical reference.

## Materials and methods

This meta-analysis has been registered in the International Prospective Register of Systematic Reviews (PROSPERO) and was carried out in accordance with the PRISMA (Preferred Reporting Items for Systematic Reviews and Meta-analyses) (Supplemental Digital Content 1, http://links.lww.com/JS9/C949, Supplemental Digital Content 2, http://links.lww.com/JS9/C950)^26^ and AMSTAR (assessing the methodological quality of systematic reviews) (Supplemental Digital Content 3, http://links.lww.com/JS9/C951)^27^ guidelines.

### Search strategy and selection criteria

**Figure 1:**
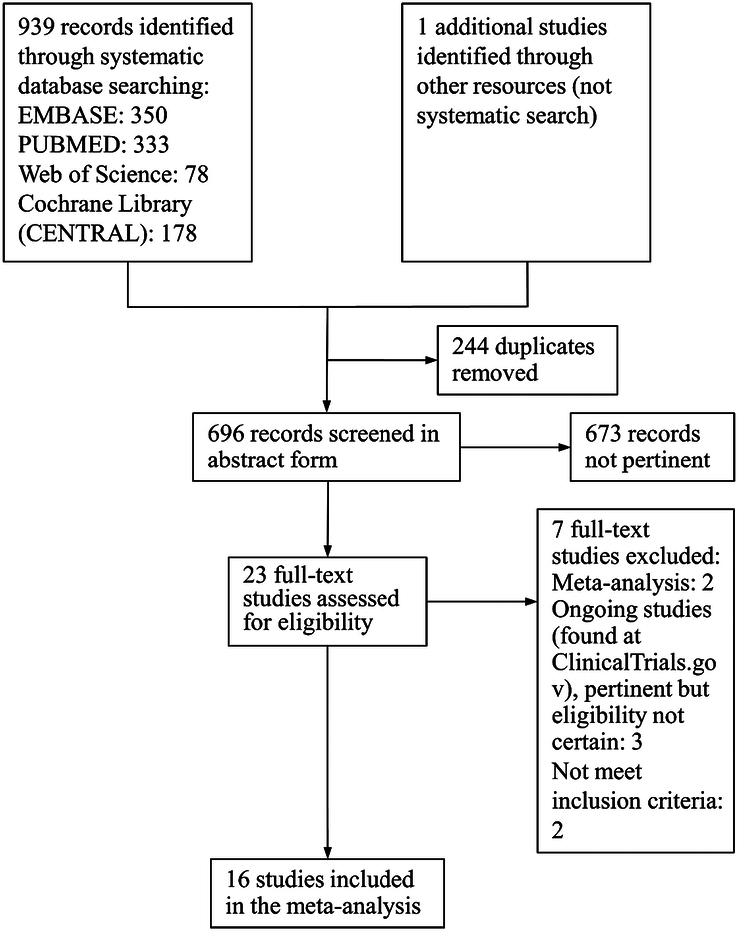
Flow chart of study selection.

We performed an extensive literature review utilizing the keywords ‘(Kataved OR S-ketamine OR Esketamine OR (S)-2-(o-chlorophenyl)-2-(methylamino)cyclohexanone OR L-Ketamine OR (-)-Ketamine OR Spravato) AND (depression OR depressive OR depressed OR mood) AND (perioperative OR anesthesia OR surgery OR perioperative),’ with the additional restrictions of ‘English or Chinese,’ ‘clinical trial’ and ‘randomized controlled trial.’ The following databases were searched: Web of Science, Cochrane Central Registry of Controlled Trials, PubMed, and Embase. Dates up until May 22, 2023, were covered by the search. All possible studies were considered, and papers that fulfilled the inclusion criteria were identified by a manual search of references. Two researchers examined the titles and abstracts of every article during the preliminary screening phase. A consensus was reached after discussing decisions that were inconsistent and disagreeing. The following were the inclusion criteria for the subsequent eligibility screening: (1) publications on human clinical trials and (2) randomized trials comparing esketamine (esketamine group) with saline or other medications (control group) for the perioperative treatment or prevention of postoperative depression. The following criteria were used to exclude articles: (1) case reports, reviews, or nonrandomized studies; (2) absence of experimental or control groups; or the inability to extract pertinent data on intriguing outcomes. The flowchart of the selection process is illustrated in Figure 1.

### Data extraction

The data were extracted from the included studies using Microsoft Excel and subsequently inserted into Review Manager (version 5.4) to conduct statistical analysis. The extracted data included the author’s name, publication year, sample size, patient’s age, sex, type of surgery, experimental grouping, type of anesthesia, timing and dose of esketamine administration, treatment in the control group, postoperative depression score, postoperative pain score, incidence of adverse reactions, and follow-up period. The primary outcome that we retrieved from the included studies was the scores of postoperative depression. The Beck Depression Inventory-II (BDI-II), Hospital Anxiety and Depression Scale (HADS), Hamilton Rating Scale for Depression Scale (HAMD-17), Self-Rating Depression Scale (SDS), and Edinburgh Postnatal Depression Scale (EPDS) were all utilized to assess postoperative depression. The efficacy of esketamine in preventing postoperative depression was evaluated at 1, 3, 7 days, and long-term (4 weeks or more) after surgery. The secondary outcome measures included postoperative pain score and adverse effects (postoperative nausea and vomiting (PONV), dizziness, drowsiness, nightmares, and dissociation). We endeavored to establish correspondence with the authors to get raw data or import data from the graphs using PlotDigitizer software when the observational data we required were not explicitly included in the article. Two trained investigators conducted independent assessments of the studies’ quality and risk of bias using the risk of bias instrument developed by the Cochrane Collaboration (Review Manager 5.4). Any disagreements were resolved through discussion, and consensus was reached. Two investigators conducted data extraction individually. A third investigator resolved any disagreements.

### Subgroup analysis

In order to determine whether various moderating variables influence the impact of esketamine on postoperative depression, subgroup analyses were carried out on the basis of multiple variables, including the absence or presence of preoperative depression, the mode of esketamine administration (single infusion vs. continuous infusion), the dosage of esketamine (low dose vs. high dose), and the type of anesthesia (spinal anesthesia vs. general anesthesia). In addition, we investigated the safety of perioperative esketamine administration in reducing postoperative depression scores. We examined the adverse effects of esketamine based on the method of esketamine usage (single infusion vs. continuous infusion), esketamine dosage (low dose vs. high dose), and anesthetic type (spinal anesthesia vs. general anesthesia).

### Summary of research results

The primary and secondary outcomes were evaluated using the Grades of Recommendation, Assessment, Development, and Evaluation (GRADE) criteria. The summary of findings for the main comparison table was created using the GRADEprofiler software.

### Statistical analysis

The meta-analysis utilized Review Manager 5.4 and Stata 17.0 software for data processing. The selection of standardized mean differences (SMD) and 95% confidence intervals (95% CI) as effect sizes for continuous variables was based on the need to account for inconsistent scales in assessing postoperative depression and postoperative pain. The risk ratio (RR) and 95% CI were selected as effect sizes for dichotomous variables. For this meta-analysis, 0.05 was used as the level of significance. The article presents results in the form of the median and interquartile range, which may be converted into the mean and standard deviation using an online data conversion formula available at math.hkbu.edu.hk/~tongt/papers/median2mean.html. When continuous variables were presented only graphically, data were obtained from the graphs by software (PlotDigitizer). Inter-study heterogeneity was estimated by the *I* ^2^ value. A fixed effects model was employed to compute the aggregated effect size when *I* ^2^<50%, indicating the lack of substantial heterogeneity. A random effects model was used when *I* ^2^≥50%, suggesting substantial heterogeneity. An investigation was carried out using subgroup analyses and leave-one-out sensitivity analysis to examine probable causes of heterogeneity.

## Results

### Search results and included study characteristics

A total of 939 possibly relevant studies were obtained, and one study was manually added by searching through references. Two hundred forty-four duplicate studies were eliminated, and 673 studies were discarded after reviewing the titles and abstracts of the literature. Seven studies were excluded from the remaining 23 on the grounds that they were ongoing, lacked pertinent data, or were the subject of meta-analyses. This meta-analysis incorporated 16 randomized controlled trials that satisfied all the inclusion criteria. Sixteen studies included a total of 2267 patients undergoing surgery^16,23–25,28–39^. Three research incorporated patients who had preoperative depression, while nine studies excluded such patients. The remaining four studies encompassed an unrestricted of patients. In six studies, esketamine was administered as a single dose, while in 10 studies, it was administered continuously. Furthermore, 10 studies employed a high dosage of esketamine (≥0.5 mg/kg), while six studies utilized a low dosage of esketamine (<0.5 mg/kg). The types of anesthesia included spinal anesthesia (five studies) and general anesthesia (11 studies). Table 1 displays the main characteristics of the studies that were included.

**Table 1:** Characteristics of included studies.

First author	Year	Study design and sample size (T/C)	Age (T/C)	Sex	Anesthesia	Surgery	Intervention and dosage (T vs. C)	Ways of esketamine injection	Timing point of intervention	Depression Measurement	Follow-up Period	Pain Measurement
Jiang *et al*.^24^	2023	RCT	32.77±5.25/	F	General anesthesia	Missed miscarriages	0.3 mg/kg i.v. ESK vs. normal saline	Single dose	Induction	EPDS	Postop days 7 and 42	VAS
		70 (35/35)	30.71±4.33									
Cheng *et al*.^28^	2022	RCT	58.0 (52.25–64.0)/	F+M	General anesthesia	Thoracic surgery	0.3 mg/kg i.v. ESK vs. normal saline and 0.125 mg/kg/h i.v. ESK vs. normal saline until 15 minutes before the end of surgery	Single dose+continuous infusion	Intraoperatively	HADS-D	Postop day 2	NRS
		77(39/38)	55.0 (51.25–61.0)									
Han *et al*.^31^	2022	RCT	31.64±3.93/	F	Spinal anesthesia	Cesarean section	0.5 mg/kg ESK+2 µg/kg Sufentanil vs. 2 µg/kg Sufentanil	PCIA	PCIA	EPDS	Postop days 3, 14 and 28	VAS
		275(122/153)	31.85±4.16									
Wang^36^	2022	RCT	28.8±6.4/28.3±5.9/27.9±6.1/	F	Spinal anesthesia	Cesarean section	0.1, 0.2, 0.4 mg/kg ESK+1.5 µg/kg Sufentanil vs. 1.5 µg/kg Sufentanil	PCIA	PCIA	EPDS	Postop days 7 and 42	VAS
		156 (38/40/39/39)	29.1±5.5									
Shen *et al*.^33^	2023	RCT	28.9±3.9/	F	Spinal anesthesia	Cesarean section	0.25 mg/kg i.v. ESK vs. normal saline	Single dose	5 min after clamping the neonatal umbilical cord	EPDS	Postop days 7, 14 and 28	NRS
		202(102/100)	29.6±3.9									
Wang^35^	2023	RCT	28.3±4.9/	F	Spinal anesthesia	Cesarean section	0.2 mg/kg i.v. ESK vs. normal saline	Single dose	10 mins after clamping the neonatal umbilical cord	EPDS	Postop days 7 and 42	
		115(58/57)	27.9±4.1									
Liu^16^	2019	RCT	46.6±8.2/	F	General anesthesia	Modified radical mastectomy	0.125 mg/kg i.v. ESK vs. normal saline	Single dose	Intraoperatively (before skin incision)	HAMD-17	Postop days 3, 7, 30, and 90	VAS
		201(101/100)	48.0±10.2									
Gan *et al*.^25^	2023	RCT	54.0±11.7/	F+M	General anesthesia	Thoracoscopic lung cancer surgery	0.1 mg/kg i.v. ESK vs. normal saline, 0.1 mg/kg/h i.v. ESK vs. normal saline until the end of surgery, and PCIA 1 mg/kg ESK+2 µg/kg Sufentanil vs. 2 µg/kg Sufentanil	Single dose+continuous infusion+PCIA	Intraoperative+PCIA	BDI-II	Postop days 2, 30 and 90, and at discharge	VAS
		156(78/78)	53.8±12.1									
Yu *et al*.^38^	2022	RCT	46±10/	F	General anesthesia	Modified radical mastectomy	0.5 mg/kg i.v. ESK vs. normal saline, 0.25 mg/kg i.v. ESK vs. 0.25 µg/kg i.v. Sufentanil when necessary, and PCIA 1 mg/kg ESK vs. 2 µg/kg Sufentanil	Single dose+PCIA	Induction+PCIA	HADS-D	At discharge	NRS
		136 (68/68)	48±9									
Su *et al*.^34^	2023	RCT	54.56±11.6/	F + M	General anesthesia	Thoracoscopic lung cancer surgery	1.2 mg/kg ESK+1 µg/kg Sufentanil vs. 1.5 µg/kg Sufentanil	PCIA	PCIA	SDS	Postop days 1, 2, 3, and 7	VAS
		67 (33/34)	56.08±10.09									
Gao *et al*.^30^	2022	RCT	26.3±4.6/	F	General anesthesia	Artificial abortion	0.25 mg/kg i.v. ESK vs. 0.1 µg/kg i.v. Sufentanil	Single dose	Induction	SDS	Postop day 3	VAS
		159 (80/79)	26.5±4.7									
Wang^37^	2022	RCT	58.83±10.94/	F+M	General anesthesia	Thoracoscopic lobectomy	0.2 mg/kg i.v. ESK vs. normal saline and 0.12 mg/kg/h i.v. ESK vs. normal saline until 30 minutes before the end of surgery	Single dose+continuous infusion	Intraoperatively	HADS-D	Postop day 1	VAS
		58 (29/29)	58.34±9.14									
Qiu *et al*.^23^	2022	RCT	43 (32–49)/	F	General anesthesia	Gynecological laparoscopic surgery	0.3 mg/kg/h i.v. ESK vs. normal saline	Continuous infusion	Intraoperatively	HADS-D	Postop days 1 and 3	NRS
		183 (92/91)	45 (35–49)									
Zhang Z^39^	2023	RCT	35.7±9.3/	F+M	General anesthesia	Bowel resection	0.25 mg/kg i.v. ESK vs. normal saline and 0.12 mg/kg/h i.v. ESK vs. normal saline for more than 30 minutes	Single dose+continuous infusion	Induction+intraoperatively	HAMD-17	Postop days 1, 3, 7 and 30	NRS
		120 (60/60)	35.2±10.9									
Min *et al*.^32^	2023	RCT	73.9±6.5/	F+M	General anesthesia	Total hip replacement	2.5 mg/kg ESK vs. 2.5 µg/kg Sufentanil	PCIA	PCIA	HADS-D	Postop days 3, 7 and 30	VAS
		132 (67/65)	73.9±6.1									
Gao *et al*.^29^	2022	RCT	37.2±1.5/	F	Spinal anesthesia	Cesarean section	0.5 mg/kg i.v. ESK vs. normal saline	Single dose	After clamping the neonatal umbilical cord	EPDS	Postop days 1 and 5	
		160 (80/80)	37.3±1.5									

BDI-II, Beck Depression Inventory-II; EPDS, Edinburgh Postnatal Depression Scale; HADS-D, Hospital Anxiety And Depression Scale; HAMD-17, Hamilton Rating Scale for Depression Score; NRS, Numerical rating scale; PCIA, patient-controlled intravenous analgesia; RCT, randomized controlled trial; SDS, Self-Rating Depression Scale; VAS, visual analog scale.

### Risk of bias assessment

**Figure 2:**
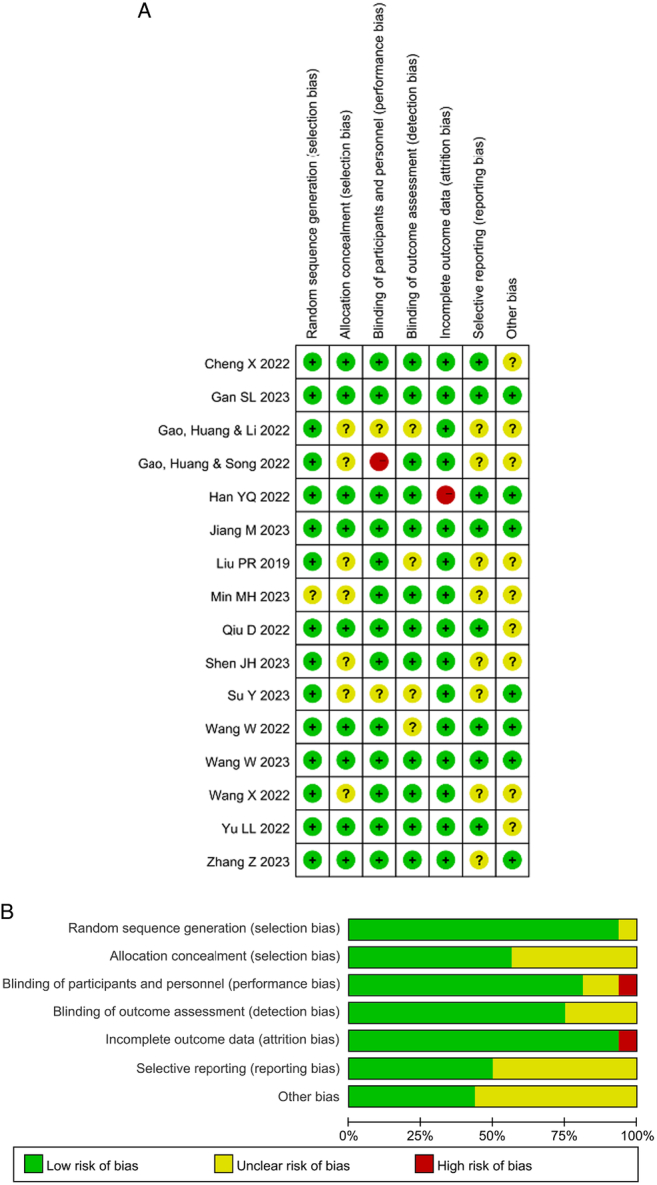
Risk of bias assessment. (A) Risk of bias for individual studies. (B) The summary of the risk of bias.

The Cochrane Handbook was utilized to evaluate the risk of bias in the studies that were included, as depicted in Figure 2. The method of generating the random sequence was described in every study except one, which failed to describe the randomization process. Nine research documented the methods used for allocation concealment. Thirteen studies detailed precise procedures for ensuring that both the participants and the investigators were blinded. In 12 studies, particular techniques for achieving blinding in outcome evaluation were described. An ‘unclear risk of bias’ was assigned to the remaining studies that failed to address blinding and allocation concealment. One study, which failed to blind the investigators, was categorized as ‘high risk’ due to the potential influence of the absence of concealment on the study’s outcome. The absence of study protocols in eight of the included studies resulted in an ‘unclear risk of bias’ assessment. Despite a loss to follow-up in eight studies, the number of participants who were lost to follow-up did not show a significant difference between the esketamine and comparison groups, and it did not have an impact on the outcomes. Consequently, we categorized this particular element of the incomplete outcome data in these studies as having a ‘low risk of bias.’ A study reported a loss to follow-up of 7.83% (13 patients) in the control group and 24.22% (39 patients) in the intervention group. As a result, the study was determined to have a ‘high risk’ of incomplete outcome data bias. Other biases have been categorized as ‘unclear risk of bias’ in nine studies as they did not describe financial conflicts of interest, postoperative depression was not the primary outcome, or sample size calculation could not be found in the studies.

### Results of meta-analysis

**Figure 3:**
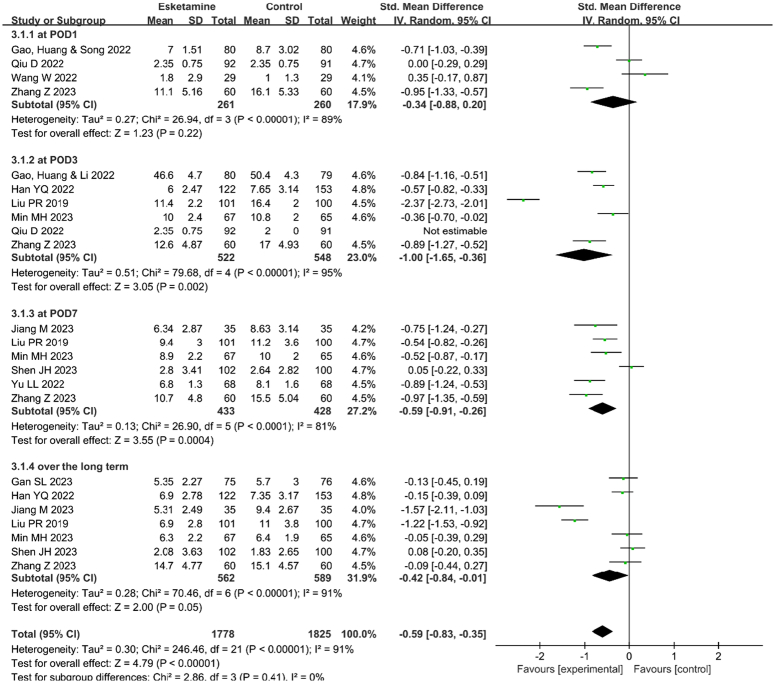
The effects of perioperative administration of esketamine on postoperative depression scores. Forest plots of the postoperative depression scores in randomized controlled trials. CI, confidence interval; df, degrees of freedom; POD, postoperative day.

The esketamine group had significantly lower postoperative depression scores compared to the control group at postoperative days (POD) 3 (SMD=−1.00, 95% CI [−1.65, −0.36], *P*=0.002, *I* ^*2*^=95%), POD 7 (SMD=−0.59, 95% CI [−0.91, −0.26], *P*=0.0004, *I* ^2^=81%), and in the long term (SMD=−0.42, 95% CI [−0.84, −0.01], *P*=0.05, *I* ^2^=91%). Postoperative depression scores on POD1 did not differ significantly between the esketamine and control groups (SMD=−0.34, 95% CI [−0.88, 0.20], *P*=0.22, *I* ^2^=89%), as shown in the Figure 3.

### Incidence of postoperative adverse reactions

**Figure 4:**
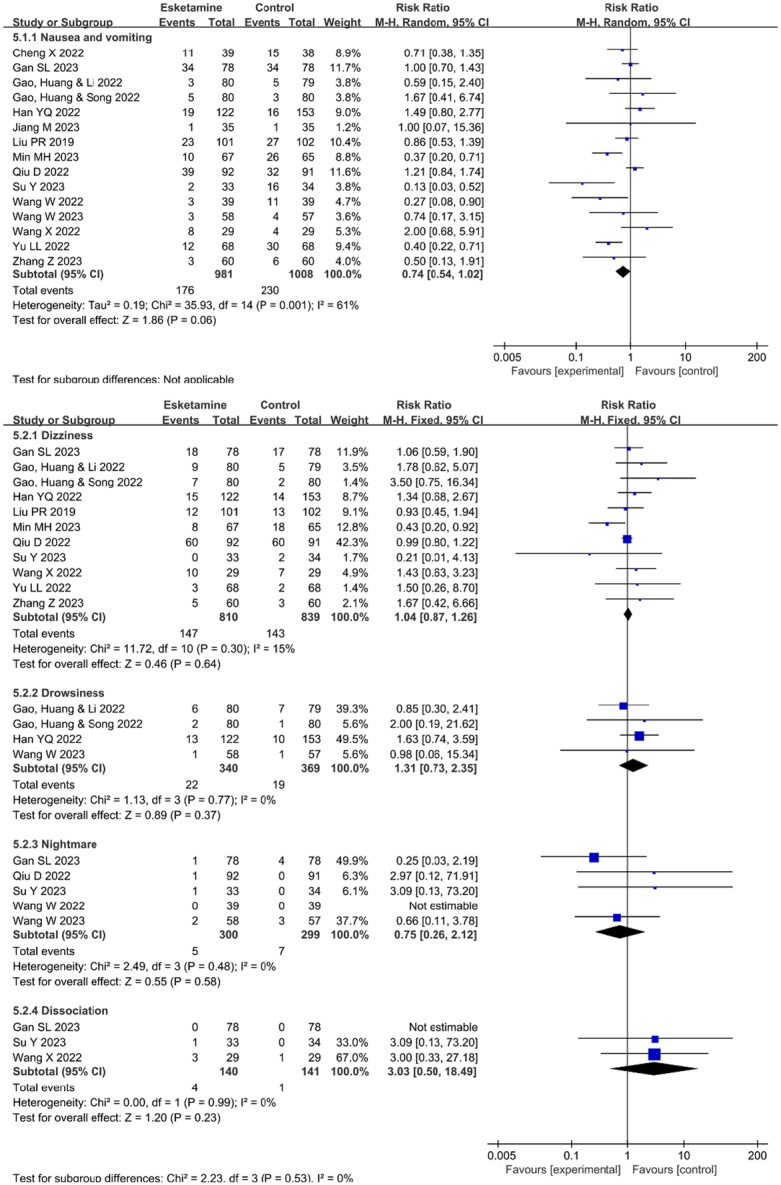
The effects of perioperative administration of esketamine on the postoperative adverse effects. Forest plots of the incidence of postoperative adverse effects in randomized controlled trials. CI, confidence interval; df, degrees of freedom.

Fifteen studies documented the occurrence of PONV. The incidence of PONV did not differ significantly between the esketamine and control groups (RR=0.74, 95% CI [0.54, 1.02], *P*=0.06, *I* ^2^=61%). The outcome is displayed in Figure. 4. Furthermore, we conducted a subgroup analysis on the method of administration, dosage, and type of anesthetic for esketamine. Our findings indicate that these factors have no impact on the occurrence of PONV (Supplementary Figures 1–3, Supplemental Digital Content 4, http://links.lww.com/JS9/C952).

Eleven studies documented the occurrence of dizziness (RR=1.04, 95% CI [0.87, 1.26], *P*=0.64, *I* ^2^=15%); four studies reported drowsiness (RR=1.31, 95% CI [0.73, 2.35], *P*=0.37, *I* ^2^=0%); five studies documented nightmare (RR=0.75, 95% CI [0.26, 2.12], *P*=0.58, *I* ^2^=0%); and three studies documented dissociation (RR=3.03, 95% CI [0.50, 18.49], *P*=0.23, *I* ^2^=0%). The incidence of the aforementioned adverse effects did not differ significantly between the esketamine and control groups. The outcomes are displayed in Figure 4.

### Postoperative pain score

**Figure 5:**
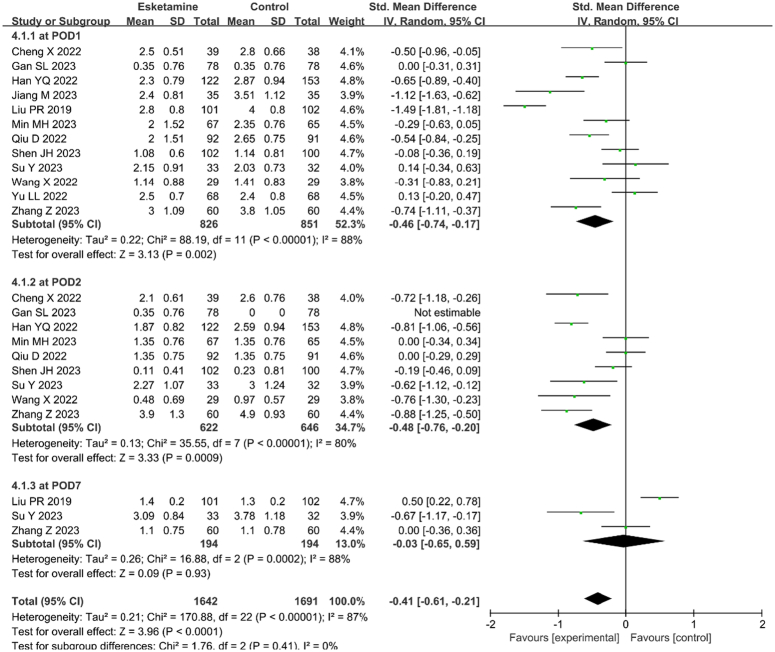
The effects of perioperative administration of esketamine on postoperative pain scores. Forest plots of postoperative pain intensity in randomized controlled trials. CI, confidence interval; df, degrees of freedom.

The esketamine group exhibited markedly reduced pain scores at 24 h (SMD=−0.46, 95% CI [−0.74, −0.17], *P*=0.002, *I* ^2^=88%) and 48 h (SMD=−0.48, 95% CI [−0.76, −0.20], *P*=0.0009, *I* ^2^=80%) postoperatively compared to the control group. At POD7, the pain scores of the esketamine and control groups did not differ significantly (SMD=−0.03, 95% CI [−0.65, 0.59], *P*=0.93, *I* ^2^=88%). The results are displayed in Figure 5.

### Subgroup analysis

The majority of the outcomes of this meta-analysis showed substantial heterogeneity among the included studies. We performed subgroup analyses of ‘postoperative depression scores on POD3’ according to the presence or absence of preoperative depression, esketamine administration method, dose, and type of anesthesia. Esketamine significantly reduced postoperative depression scores on the POD3 in both the high-dose (SMD=−0.50, 95% CI [−0.70, −0.30], *P*<0.00001, *I* ^2^=0%) and low-dose (SMD=−1.37, 95% CI [−2.34, −0.39], *P*=0.006, *I* ^2^=96%) groups, in both the with preoperative depression (SMD=−1.63, 95% CI [−3.08, −0.18], *P*=0.03, *I* ^2^=97%) and no preoperative depression (SMD=−0.59, 95% CI [−0.84, −0.35], *P*<0.00001, *I* ^2^=50%) groups, in both the single infusion (SMD=−1.60, 95% CI [−3.10, −0.10], *P*=0.04, *I* ^2^=97%) and continuous infusion (SMD=−0.60, 95% CI [−0.86, −0.33], *P*<0.0001, *I* ^2^=53%) groups, and in both the spinal (SMD=−0.57, 95% CI [−0.82, −0.33], *P*<0.00001) and general anesthesia (SMD=−1.11, 95% CI [−1.95, −0.28], *P*=0.009, *I* ^2^=96%) groups (Supplementary Figures 4–7, Supplemental Digital Content 4, http://links.lww.com/JS9/C952).

The long-term therapeutic effect of drugs is more concerned than the short-term effect. We performed subgroup analyses of the long-term postoperative depression scores. It was found that esketamine significantly reduced long-term postoperative depression scores in patients with preoperative depression (SMD=−0.95, 95% CI [−1.81, −0.08], *P*=0.03, *I* ^2^=93%), or undergoing general anesthesia (SMD=−0.59, 95% CI [−1.18, −0.01], *P*=0.05, *I* ^2^=93%) (Supplementary Figures 8–11, Supplemental Digital Content 4, http://links.lww.com/JS9/C952).

### Sensitivity analysis

A leave-one-out sensitivity analysis was conducted to examine the stability and sources of heterogeneity in the outcomes of this meta-analysis. We observed that all of the outcomes were stable and dependable except for the postoperative depression over the long term and PONV (Supplementary Figures 12–14, Supplemental Digital Content 4, http://links.lww.com/JS9/C952).

### Summary of findings for the main comparison

This meta-analysis used the GRADE system to assess the strength of the evidence for the main and secondary findings. The majority of the 16 studies considered had a mostly low or very low quality of evidence, primarily due to heterogeneity, imprecise outcomes, and a possibility of bias. The outcomes are displayed in Supplementary Table 1 (Supplemental Digital Content 5, http://links.lww.com/JS9/C953).

## Discussion

Our meta-analysis showed that perioperative use of esketamine was effective in reducing postoperative depression scores, and this effect persisted even beyond 1 month after surgery. Esketamine can effectively relieve the postoperative pain scores on the POD1 and POD2 and does not increase the incidence of adverse effects such as PONV, dizziness, drowsiness, nightmares, and dissociation.

Esketamine significantly decreased postoperative depression scores, and this effect persisted for a long time following the surgery. Our prior meta-analysis^22^ demonstrated that esketamine effectively decreased the occurrence of PPD. Furthermore, even 42 days after childbirth, the incidence of PPD in the group that received esketamine was still lower than in the control group, which is consistent with the findings of the present meta-analysis. Nevertheless, the incongruous findings in this meta-analysis should not be disregarded. Shen *et al.* ^33^ discovered that the single infusion of low-dose esketamine (0.25 mg/kg) during cesarean section did not result in a reduction in the occurrence of PPD at the 1, 2, or 4-week postpartum intervals. One possible explanation is that the recruited patients may have been in better general health, with PPD rates much lower than reported rates of 10–22%^40^. One week following surgery, the esketamine group had a PPD prevalence of 3.9% (four patients), whereas the control group had a PPD prevalence of 2% (two patients). Two weeks postpartum, the PPD prevalence was 2% (two patients) in the esketamine group and 1% (one patient) in the control group. Additional randomized controlled studies are necessary to ascertain the efficacy of perioperative esketamine administration in preventing postoperative depression.

The precise mechanism underlying the antidepressant action of esketamine remains uncertain. Evidence from preclinical research has indicated that brain-derived neurotrophic factor and mammalian target of rapamycin complex 1 have a significant impact on the antidepressant properties of esketamine^18^. Evidence from clinical trials indicated that esketamine reduces postoperative depression by correlating with elevated blood brain-derived neurotrophic factor levels^17^. Yang *et al*.^41^ provided evidence that mammalian target of rapamycin complex 1 signaling contributes to esketamine’s antidepressant effects. Additionally, esketamine has the potential to alleviate depression by regulating the immune system and reducing inflammation. According to a recent study by Jiang *et al.* ^24^, esketamine successfully alleviated postoperative depression in patients who experienced a missed abortion while also reducing the production of proinflammatory cytokines interleukin-6, interleukin-1β, and tumor necrosis factor-α. Furthermore, esketamine is progressively being utilized as an adjuvant to perioperative analgesia^42–45^. Depression and pain have a close relationship and can cause each other^46–48^. Esketamine showed efficacy in improving postoperative pain scores on both POD1 and POD2 in this trial. This result is consistent with the conclusion reached in a previous meta-analysis that esketamine effectively reduced pain scores 4, 12, 24, and 48 h after cesarean section^49^. Perfect postoperative analgesia can effectively reduce the postoperative depression score, improve postoperative recovery, and shorten the length of hospital stay^48,50^.

To investigate the most effective dosage of esketamine for preventing postoperative depression, we conducted a subgroup analysis explicitly focusing on the different doses of esketamine administered. The short-term and long-term effects of esketamine on postoperative depression were analyzed. In both the high-dose (≥0.5 mg/kg) and low-dose (<0.5 mg/kg) groups, esketamine considerably decreased both the short-term and long-term postoperative depression scores. A recent study conducted by Wang and colleagues examined the effects of varying dosages of esketamine (0.1, 0.2, and 0.4 mg/kg) on antidepressant efficacy. The results indicated that the doses of 0.2 and 0.4 mg/kg of esketamine showed superior antidepressant efficacy compared to the dose of 0.1 mg/kg and the control group^36^. Furthermore, Shen *et al*.^33^ discovered that administering a single dose of 0.25 mg/kg esketamine did not yield any significant benefits in terms of postoperative depression scores when compared to the control group. Unfortunately, there is a limited number of clinical trials investigating the impact of various dosages of esketamine administered during the perioperative period on postoperative depression scores, so we cannot obtain the optimal dose of esketamine to improve postoperative depression through the current data. The range of esketamine doses in this meta-analysis varied from 0.1 to 2.5 mg/kg, with the most often administered dose being 0.5 mg/kg. There is a common belief that the side effects become more severe as the dosage increases. However, we have discovered that the administration of large amounts of esketamine during the perioperative period does not lead to a higher occurrence of adverse effects after surgery^32^. Additional randomized, double-blind trials that directly compare various doses are necessary to ascertain the most effective dose of esketamine for preventing postoperative depression.

The safety and effectiveness of esketamine in the prevention of postoperative depression may be influenced by the method of administration. Hence, we performed a subgroup analysis to examine the various modes of administering esketamine. The perioperative administration of esketamine, whether through a single administration or continuous administration, was an effective method for preventing postoperative depression. Furthermore, it did not lead to an increased occurrence of postoperative side effects. Guo *et al*.’s^1^ meta-analysis revealed that administering ketamine during the perioperative period led to a higher occurrence of side effects such as PONV, dizziness, headache, and hallucinations. However, when ketamine was continuously infused, it effectively reduced the incidence of these adverse reactions while still maintaining its antidepressant effect, as compared to a single infusion of ketamine. Our investigation revealed that esketamine did not contribute to a higher occurrence of adverse effects following surgery. Consequently, esketamine exhibited a lower incidence of adverse effects compared to ketamine, aligning with prior findings^15–17^. Future studies need to directly compare the effects of a single infusion of esketamine with continuous infusion, as more well-designed research in this area is needed.

Does a general anesthetic affect the efficacy of perioperative esketamine in preventing postoperative depression? A subgroup analysis was conducted based on the type of anesthetic. In both the spinal anesthesia and general anesthesia subgroups, esketamine was effective in reducing the postoperative depression scores on the POD3. In the long term, esketamine was more effective in improving postoperative depression in the general anesthesia subgroup than in the spinal anesthesia subgroup. The possible reason may be that there were few studies in the subgroup of long-term postoperative spinal anesthesia, only two studies were included^31,33^. In summary, the effectiveness of esketamine in preventing postoperative depression was not impacted by the use of general anesthetics. Esketamine cannot only effectively prevent postoperative depression but also effectively reduce postoperative depression scores in patients with preoperative depression. Regardless of the presence or absence of preoperative depression, esketamine was effective in reducing postoperative depression scores on the POD3. However, in the reduction of long-term postoperative depression scores by esketamine, the effect of esketamine seems to be better in the subgroup with preoperative depression than in the subgroup without preoperative depression, which may be related to different types of surgery and differences in general conditions of patients. More studies are needed to investigate further the effect of the presence or absence of preoperative depression on the treatment of perioperative depression with esketamine and the prevention of postoperative depression.

Caution should be exercised when interpreting the results of this meta-analysis due to the limitations outlined below, which mainly stem from the shortcomings of the original studies. Primarily, the majority of the findings of this meta-analysis exhibited heterogeneity because of the limited sample size in most studies, variations in surgical procedures, diverse anesthetic techniques, and inconsistencies in the methods and dosages of esketamine administration. Furthermore, there were a limited number of articles (four to seven articles) on postoperative depression scores at each observation time point (POD1, POD3, POD7, and over the long term), so we did not perform subgroup analyses by type of surgery. In addition, esketamine was provided at various levels, spanning from 0.1 to 2.5 mg/kg. Hence, it is not possible to assess the ideal dosage of esketamine to prevent postoperative depression. What is more, the utilization of distinct measurement scales to evaluate postoperative depression and postoperative pain contributes to the existing heterogeneity. Notably, all the studies incorporated in this meta-analysis originated in China, thereby possibly limiting the application of the results to other nations. In 2019, intravenous esketamine was launched in China. Subsequently, there has been a progressive increase in studies conducted in China on the use of esketamine during the perioperative period. To summarize, additional randomized controlled trials with substantial sample sizes are required to substantiate further the efficacy of esketamine in lowering postoperative depression scores and alleviating postoperative pain.

## Conclusion

This meta-analysis demonstrated that esketamine administered perioperatively reduced postoperative depression scores and that this effect persisted for an extended period. Esketamine was quite effective in reducing postoperative pain scores. Esketamine did not increase the risk of PONV, dizziness, drowsiness, nightmares, or dissociation when compared to the control group. In future clinical practice, it remains a major challenge how to achieve the best effect of preventing postoperative depression with the lowest esketamine dose.

## Ethical approval

This is a meta-analysis and does not involve ethical approval.

## Consent

This is a meta-analysis and does not involve patient privacy.

## Source of funding

This project was sponsored by the Nanjing Health Bureau Medical Science and Technology Development Foundation (No. YKK23153).

## Author contribution

Y.Z.W., M.J.M., and M,J, have access to all the study data and assume responsibility for the data integrity. H.M.Y., X.W., and S.W.F. contributed to the study’s conception and design. Q.Q.L., Q.L., and X.W. contributed to the data collection and collation. Y.Z.W., M.J.M., and M.J. performed the statistical analysis and manuscript writing. S.W.F., X.W., and H.M.Y. supervised the study. All authors approved the final manuscript.

## Conflicts of interest disclosure

There are no conflicts of interest.

## Research registration unique identifying number (UIN)

This meta-analysis has been registered in the International Prospective Register of Systematic Reviews (PROSPERO) (No. CRD42023433742).

## Guarantor

The corresponding authors (Shanwu Feng, Xian Wang, and Hongmei Yuan) are responsible for this article.

## Data availability statement

Correspondence and material requests should be addressed to Shanwu Feng, Xian Wang, or Hongmei Yuan.

## Provenance and peer review

Not commissioned, externally peer-reviewed.

## Supplementary Material
