# Supplementary material for: Efficacy and safety of perioperative application of esketamine on postoperative depression: a meta-analysis of randomized controlled studies
Source: Int J Surg. 2024 Jun 27;111(1):1191–202. doi: 10.1097/JS9.0000000000001870 (PMC11745698; doi:10.1097/JS9.0000000000001870)
Supplement: Supplementary file 1 [file js9-111-1191-s001.docx]

| **Section and Topic** | **Item #** | **Checklist item** | **Location where item is reported** |
| --- | --- | --- | --- |
| **TITLE** | | |  |
| Title | 1 | Efficacy and safety of perioperative application of esketamine on postoperative depression: A meta-analysis of randomized controlled studies | Page 1 |
| **ABSTRACT** | | |  |
| Abstract | 2 | Postoperative depression, a prevalent psychological complication following surgical procedures, has a profound impact on patients' postoperative rehabilitation and overall quality of life. Preventing postoperative depression is of significant value because conventional antidepressants have a slow onset of action. Esketamine showed prompt and sustained antidepressant efficacy. Nevertheless, the safety and effectiveness of perioperative esketamine in preventing postoperative depression are still unknown. The purpose of this meta-analysis was to assess the safety and effectiveness of perioperative intravenous esketamine in relation to its ability to prevent postoperative depression. The included studies were randomized controlled trials. The primary outcome assessed is the postoperative depression scores. Postoperative pain ratings and adverse effects constituted secondary outcomes. A total of 16 studies encompassed 1161 patients who received esketamine intervention, whereas 1106 patients served as controls. Esketamine was efficacious in reducing postoperative depression scores when administered perioperatively, and the esketamine group maintained a lower postoperative depression score than the control group more than four weeks after surgery. Esketamine effectively alleviated postoperative pain scores without increasing the occurrence of postoperative nausea and vomiting, dizziness, drowsiness, nightmares, and dissociation. In summary, the administration of esketamine during the perioperative has the potential to decrease postoperative depression and pain scores without increasing the incidence of adverse effects. | Page 1 |
| **INTRODUCTION** | | |  |
| Rationale | 3 | Postoperative depression is a common psychological issue that occurs in individuals after surgery. It is characterized by psychological stress reactions, including depression and anxiety. The previous studies demonstrate that it has a significant impact on the effectiveness of postoperative rehabilitation and the general well-being of patients. Postoperative depression is associated with postoperative pain, cognitive deterioration, extended inpatient length, and potentially reduced survival time. More than 24% of surgical patients have reported experiencing perioperative depression, with a proportion of nearly 47% in patients having cardiac surgery. Unfortunately, there were few options for treating the emergence of depressive symptoms during the perioperative period or proactively preventing the occurrence of postoperative depression by administering antidepressants beforehand. | Page 2 |
| Objectives | 4 | Esketamine has nearly similar pharmacological characteristics as ketamine which has demonstrated a prompt, sustained, and efficacious impact on treatment-resistant depression and major depressive disorder, but with fewer side effects. Despite the growing body of research in recent years concerning the perioperative use of esketamine, its safety and effectiveness in treating postoperative depression in surgical patients remain unknown. Therefore, we conducted a meta-analysis on the perioperative use of esketamine for the prevention or treatment of postoperative depression and its associated adverse effects to provide a clinical reference. | Pages 2-3 |
| **METHODS** | | |  |
| Eligibility criteria | 5 | The following were the inclusion criteria for the subsequent eligibility screening: (1) publications on human clinical trials and (2) randomized trials comparing esketamine (Esketamine group) with saline or other medications (Control group) for the perioperative treatment or prevent of postoperative depression. The following criteria were used to exclude articles: (1) Case reports, reviews, or non-randomized studies; (2) Absence of experimental or control groups; or Inability to extract pertinent data on intriguing outcomes. | Pages 3-4 |
| Information sources | 6 | The following databases were searched: Web of Science, Cochrane Central Registry of Controlled Trials, PubMed, and Embase. All possible studies were considered, and papers that fulfilled the inclusion criteria were identified by a manual search of references. | Page 3 |
| Search strategy | 7 | We performed an extensive literature review utilizing the keywords "(Kataved OR S-ketamine OR Esketamine OR (S)-2-(o-chlorophenyl)-2-(methylamino)cyclohexanone OR L-Ketamine OR (-)-Ketamine OR Spravato) AND (depression OR depressive OR depressed OR mood) AND (perioperative OR anesthesia OR surgery OR perioperative)", with the additional restrictions of "English or Chinese", "clinical trial" and "randomized controlled trial". | Page 3 |
| Selection process | 8 | Two researchers examined the titles and abstracts of every article during the preliminary screening phase. A consensus was reached after discussing decisions that were inconsistent and disagreeing. | Page 3 |
| Data collection process | 9 | Two investigators conducted data extraction individually. A third investigator resolved any disagreements. | Page 4 |
| Data items | 10a | The extracted data included the author's name, publication year, sample size, patient's age, gender, type of surgery, experimental grouping, type of anesthesia, timing and dose of esketamine administration, treatment in the control group, postoperative depression score, postoperative pain score, incidence of adverse reactions, and follow-up period.  The primary outcome that we retrieved from the included studies was the scores of postoperative depression. The Beck Depression Inventory-II (BDI-II), Hospital Anxiety and Depression Scale (HADS), Hamilton Rating Scale for Depression Scale (HAMD-17), Self-rating Depression Scale (SDS), and Edinburgh Postnatal Depression Scale (EPDS) were all utilized to assess postoperative depression. The efficacy of esketamine in preventing postoperative depression was evaluated at 1 day, 3 days, 7 days, and long-term (4 weeks or more) after surgery.  The secondary outcome measures included postoperative pain score and adverse effects (postoperative nausea and vomiting, dizziness, drowsiness, nightmare, and dissociation).  We endeavored to establish correspondence with the authors to get raw data or import data from the graphs using PlotDigitizer software when the observational data we required were not explicitly included in the article. | Page 4 |
|  | 10b | We endeavored to establish correspondence with the authors to get raw data or import data from the graphs using PlotDigitizer software when the observational data we required were not explicitly included in the article. | Page 4 |
| Study risk of bias assessment | 11 | Two trained investigators conducted independent assessments of the studies' quality and risk of bias using the risk of bias instrument developed by the Cochrane Collaboration (Review Manager 5.4). Any disagreements were resolved through discussion, and consensus was reached. | Page 4 |
| Effect measures | 12 | The selection of standardized mean differences (SMD) and 95% confidence intervals (95% CI) as effect sizes (ES) for continuous variables was based on the need to account for inconsistent scales in assessing postoperative depression and postoperative pain. The risk ratio (RR) and 95% confidence interval (CI) were selected as effect sizes for dichotomous variables. | Page 5 |
| Synthesis methods | 13a | We included randomized controlled trials comparing esketamine (Esketamine group) with saline or other medications (Control group) for the perioperative treatment or prevent of postoperative depression. Of the 16 included studies, 2 studies compared esketamine with sufentanil, and the rest compared esketamine with saline. | Page 3 and table 1 |
|  | 13b | The article presents results in the form of the median and interquartile range, which may be converted into the mean and standard deviation using an online data conversion formula available at math.hkbu.edu.hk/~tongt/papers/median2mean.html. When continuous variables were presented only graphically, data were obtained from the graphs by software (PlotDigitizer). | Pages 5-6 |
|  | 13c | Review Manager 5.4 was used to used to produce forest graphs. Stata 17.0 software was used for leave-one-out sensitivity analysis to examine probable causes of heterogeneity. for data processing. The summary of findings for the main comparison table was created using the GRADEprofiler software. | Pages 5-6 |
|  | 13d | The meta-analysis utilized Review Manager 5.4 and Stata 17.0 software for data processing. Inter-study heterogeneity was estimated by the I2 value. A fixed effects model was employed to compute the aggregated effect size when I2 < 50%, indicating the lack of substantial heterogeneity. A random effects model was used when I2 ≥ 50%, suggesting substantial heterogeneity. | Page 6 |
|  | 13e | An investigation was carried out using subgroup analyses and leave-one-out sensitivity analysis to examine probable causes of heterogeneity. | Pages 5-6 |
|  | 13f | We used leave-one-out sensitivity analysis to assess robustness of the synthesized results. | Page 6 |
| Reporting bias assessment | 14 | A study reported a loss to follow-up of 7.83% (13 patients) in the control group and 24.22% (39 patients) in the intervention group. As a result, the study was determined to have a "high risk" of incomplete outcome data bias. | Page 7 |
| Certainty assessment | 15 | The primary and secondary outcomes were evaluated using the Grades of Recommendation, Assessment, Development, and Evaluation (GRADE) criteria. The summary of findings for the main comparison table was created using the GRADEprofiler software. | Page 5 |
| **RESULTS** | | |  |
| Study selection | 16a | Figure 1 in our meta-analysis presents the retrieval and screening process of articles. | Page 6 and figure 1 |
|  | 16b | There were no studies that appeared to meet the inclusion criteria, but which were excluded. | Page 6 and figure 1 |
| Study characteristics | 17 | Supplementary Table 1 in our meta-analysis presents the each included study and its characteristics. | Page 9 and supplementary table 1 |
| Risk of bias in studies | 18 | The Cochrane Handbook was utilized to evaluate the risk of bias in the studies that were included, as depicted in Figure 2. | Page 6 and figure 2 |
| Results of individual studies | 19 | Figures 3-5, supplementary figures 1-14 and supplementary table 1 in our meta-analysis presents the results. | Pages 7-9 and supplementary figures 1-14 and table 1 |
| Results of syntheses | 20a | The primary and secondary outcomes were evaluated using the Grades of Recommendation, Assessment, Development, and Evaluation (GRADE) criteria. The summary of findings for the main comparison table was created using the GRADEprofiler software. | Page 9 and supplementary table 1 |
|  | 20b | Figures 3-5 and supplementary figures 1-14 present results of all statistical syntheses conducted. | Pages 7-9, figures 3-5 and supplementary figures 1-14 |
|  | 20c | The majority of the outcomes of this meta-analysis showed substantial heterogeneity among the included studies. We performed subgroup analyses according to the presence or absence of preoperative depression, esketamine administration method, dose, and type of anesthesia. | Pages 8-9 |
|  | 20d | Supplementary figures 12-14 present the results of leave-one-out sensitivity analysis to assess the robustness of the synthesized results. | Page 9 and supplementary figures 12-14 |
| Reporting biases | 21 | A study reported a loss to follow-up of 7.83% (13 patients) in the control group and 24.22% (39 patients) in the intervention group. As a result, the study was determined to have a "high risk" of incomplete outcome data bias. | Pages 6-7 |
| Certainty of evidence | 22 | This meta-analysis used the GRADE system to assess the strength of the evidence for the main and secondary findings. The majority of the sixteen studies considered had a mostly low or very low quality of evidence, primarily due to heterogeneity, imprecise outcomes, and a possibility of bias. The outcomes are displayed in Supplementary Table 1. | Page 9 and supplementary table 1 |
| **DISCUSSION** | | |  |
| Discussion | 23a | We interpret the meta-analysis results in the context of other evidence in the discussion section of the manuscript. | Pages 10-13 |
|  | 23b | We discussed the limitations of the evidence in the discussion section of the manuscript. | Pages 10-13 |
|  | 23c | Caution should be exercised when interpreting the results of this meta-analysis due to the limitations outlined below, which mainly stem from the shortcomings of the original studies. Primarily, the majority of the findings of this meta-analysis exhibited heterogeneity because of the limited sample size in most studies, variations in surgical procedures, diverse anesthetic techniques, and inconsistencies in the methods and dosages of esketamine administration. Furthermore, esketamine was provided at various levels, spanning from 0.1 to 2.5 mg/kg. Hence, it is not possible to assess the ideal dosage of esketamine to prevent postoperative depression. Furthermore, the utilization of distinct measurement scales to evaluate postoperative depression and postoperative pain contributes to the existing heterogeneity. Notably, all the studies incorporated in this meta-analysis originated in China, thereby possibly limiting the application of the results to other nations. In 2019, intravenous esketamine was launched in China. Subsequently, there has been a progressive increase in studies conducted in China on the use of esketamine during the perioperative period. | Pages 13-14 |
|  | 23d | In future clinical practice, it remains a major challenge how to achieve the best effect of preventing postoperative depression with the lowest esketamine dose. | Page 14 |
| **OTHER INFORMATION** | | |  |
| Registration and protocol | 24a | This meta-analysis has been registered in the International Prospective Register of Systematic Reviews (PROSPERO) (No. CRD42023433742). | Page 3 |
|  | 24b | The review protocol can be accessed in the International Prospective Register of Systematic Reviews (PROSPERO) (No. CRD42023433742). | Page 3 |
|  | 24c | Few modifications were made. | Page 3 |
| Support | 25 | This project was sponsored by the Nanjing Health Bureau Medical Science and Technology Development Foundation (No. YKK23153). | Page 14 |
| Competing interests | 26 | All authors declare that they have no conflicts of interest. | Page 14 |
| Availability of data, code and other materials | 27 | Correspondence and material requests should be addressed to Shanwu Feng, Xian Wang, or Hongmei Yuan. | Page 14 |
