# Supplementary material for: Efficacy and safety of perioperative application of esketamine on postoperative depression: a meta-analysis of randomized controlled studies
Source: Int J Surg. 2024 Jun 27;111(1):1191–202. doi: 10.1097/JS9.0000000000001870 (PMC11745698; doi:10.1097/JS9.0000000000001870)
Supplement: Supplementary file 2 [file js9-111-1191-s002.docx]

**Identification of studies via other methods**

**Identification of studies via databases and registers**

Records identified from:

Citation searching (n = 1)

etc.

Records removed *before screening*:

Duplicate records removed (n = 244)

939 records identified from:

EMBASE (n = 350)

PUBMED (n = 333)

Web of Science (n = 78)

Cochrane Library (CENTRAL n = 178)

**Identification**

Records screened

(n = 695)

Records excluded

(n = 673)

Reports not retrieved

(n = 0)

Reports sought for retrieval

(n = 1)

Reports sought for retrieval

(n = 22)

Reports not retrieved

(n = 0)

**Screening**

Reports assessed for eligibility

(n = 1)

Reports excluded (n = 0)

Reports assessed for eligibility

(n = 22)

Reports excluded:

Meta-analysis (n = 2)

Ongoing studies found at ClinicalTrials.gov), pertinent but eligibility not certain (n =3)

Not meet inclusion criteria (n =2)

Studies included in review

(n = 0)

Reports of included studies

(n = 16)

**Included**
