# Supplementary material for: Efficacy and safety of perioperative application of esketamine on postoperative depression: a meta-analysis of randomized controlled studies
Source: Int J Surg. 2024 Jun 27;111(1):1191–202. doi: 10.1097/JS9.0000000000001870 (PMC11745698; doi:10.1097/JS9.0000000000001870)

**Supplementary Figures**

**Efficacy and safety of perioperative application of esketamine on postoperative depression: A meta-analysis of randomized controlled studies**

**Supplementary Figure 1. Forest plots of subgroup analyses.** Subgroup analysis for the incidence of postoperative nausea and vomiting according to the method of esketamine administration (single administration vs. continue administration). **CI**, confidence interval. **df**, degrees of freedom.


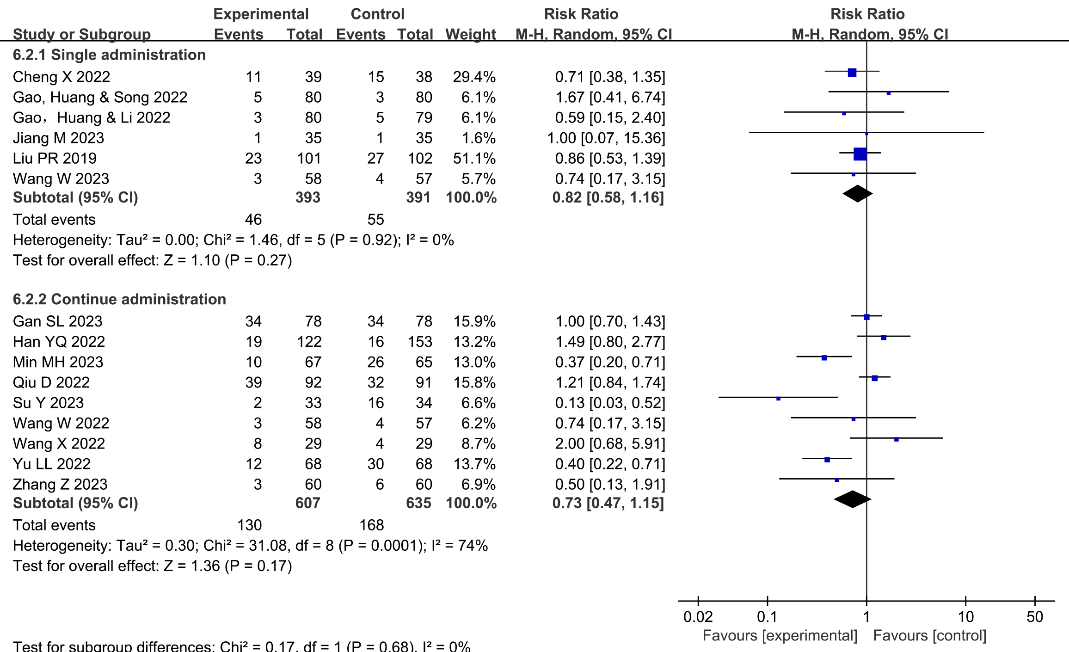


**Supplementary Figure 2. Forest plots of subgroup analyses.** Subgroup analysis for the incidence of postoperative nausea and vomiting according to the dosage of esketamine administration (low dose vs. high dose). **CI**, confidence interval. **df**, degrees of freedom.


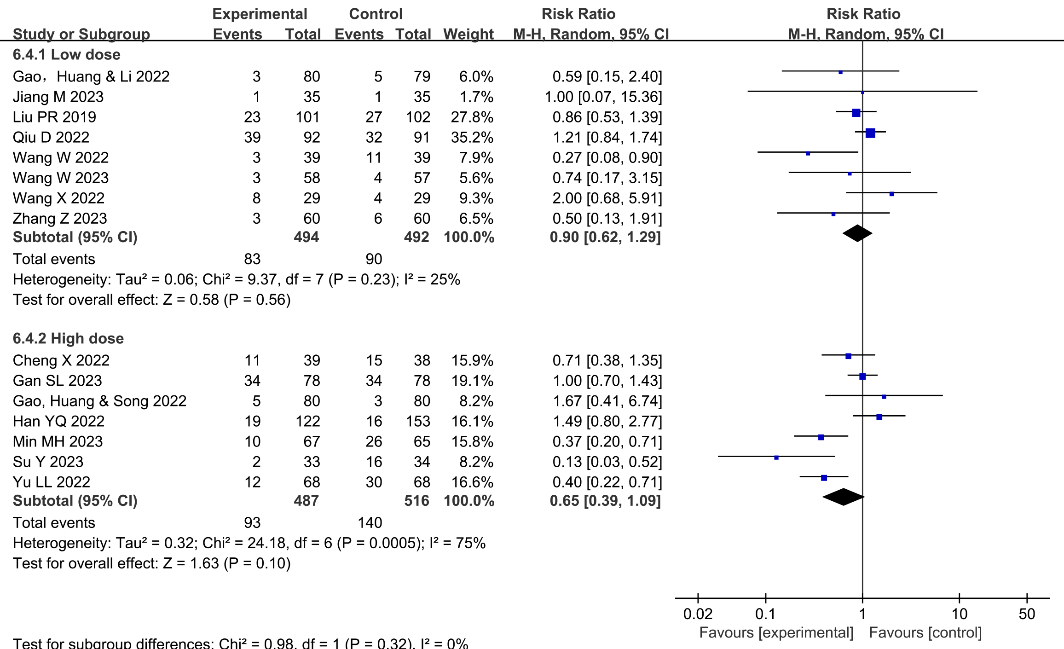


**Supplementary Figure 3. Forest plots of subgroup analyses.** Subgroup analysis for the incidence of postoperative nausea and vomiting according to the type of anesthesia (spinal anesthesia vs. general anesthesia). **CI**, confidence interval. **df**, degrees of freedom.


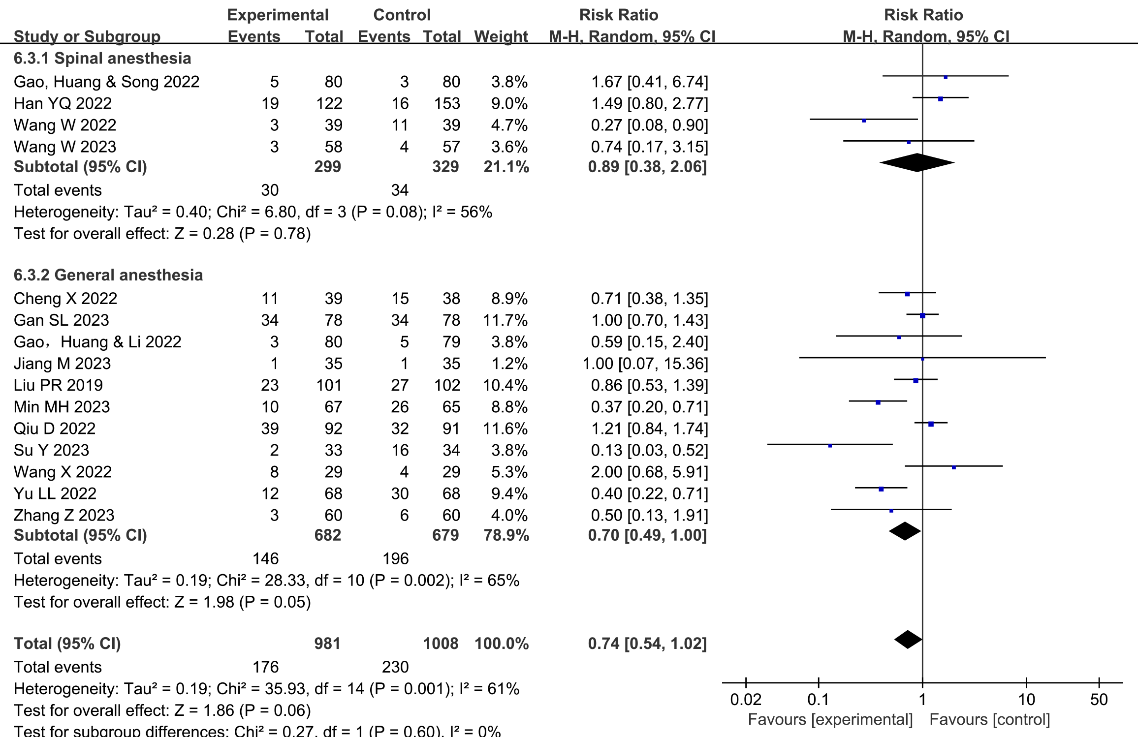


**Supplementary Figure 4. Forest plots of subgroup analyses.** Subgroup analysis for the scores of postoperative depression according to the presence or absence of preoperative depression (with preoperative depression vs. no preoperative depression) on POD 3. **CI**, confidence interval. **df**, degrees of freedom.

**
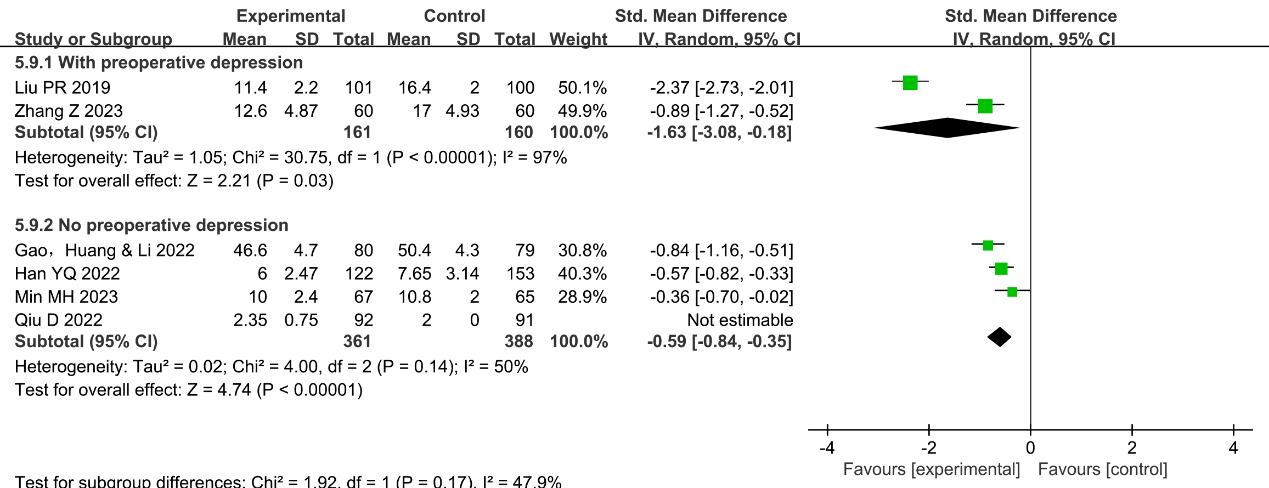
**

**Supplementary Figure 5. Forest plots of subgroup analyses.** Subgroup analysis for the scores of postoperative depression according to the method of esketamine administration (single administration vs. continue administration) on POD 3. **CI**, confidence interval. **df**, degrees of freedom.


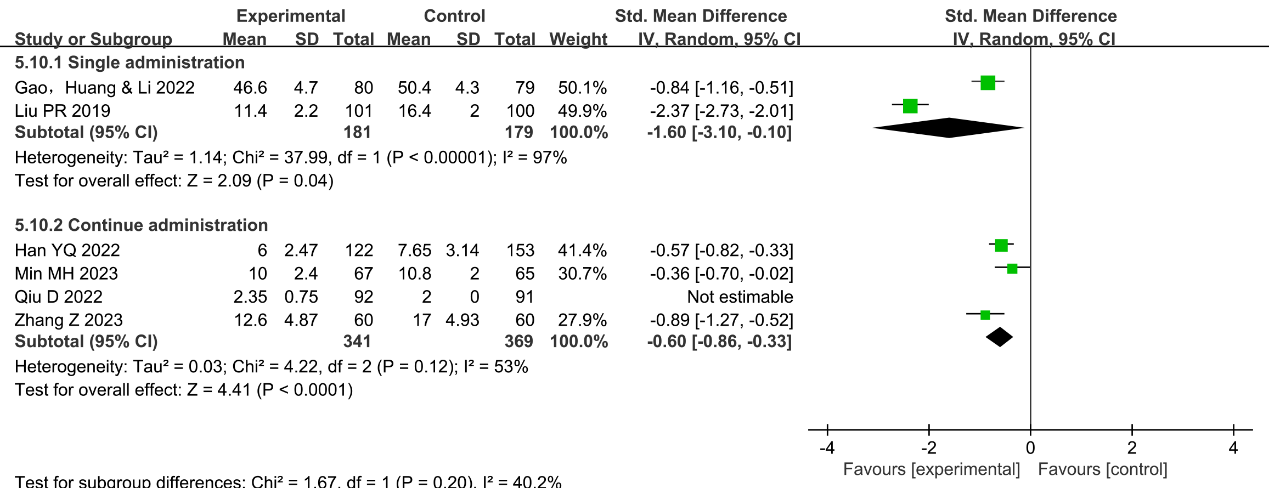


**Supplementary Figure 6. Forest plots of subgroup analyses.** Subgroup analysis for the scores of postoperative depression according to the dosage of esketamine administration (low dose vs. high dose) on POD 3. **CI**, confidence interval. **df**, degrees of freedom.

**
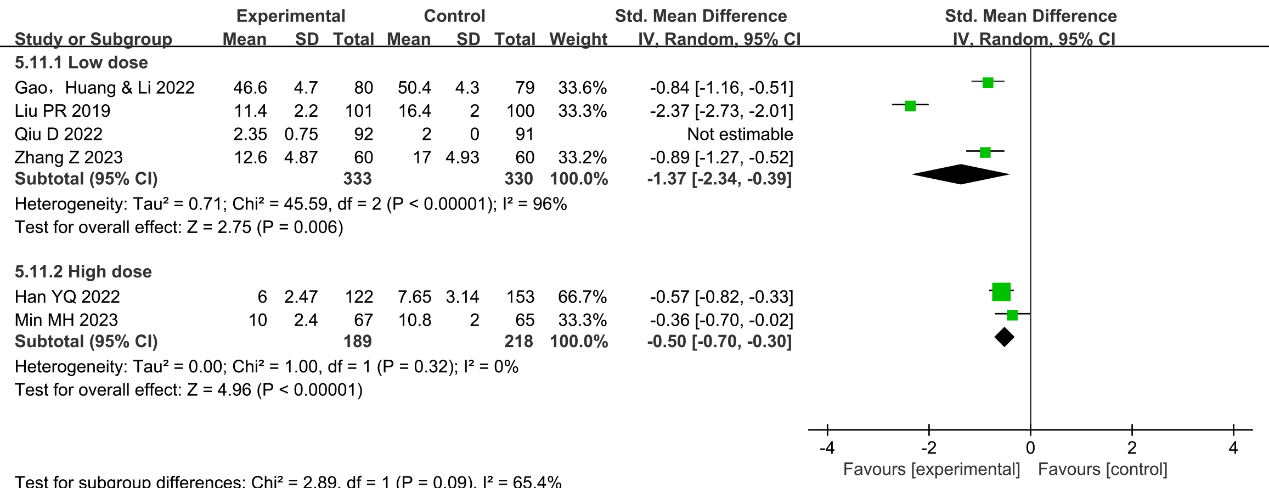
**

**Supplementary Figure 7. Forest plots of subgroup analyses.** Subgroup analysis for the scores of postoperative depression according to the type of anesthesia (spinal anesthesia vs. general anesthesia) on POD 3. **CI**, confidence interval. **df**, degrees of freedom.

**
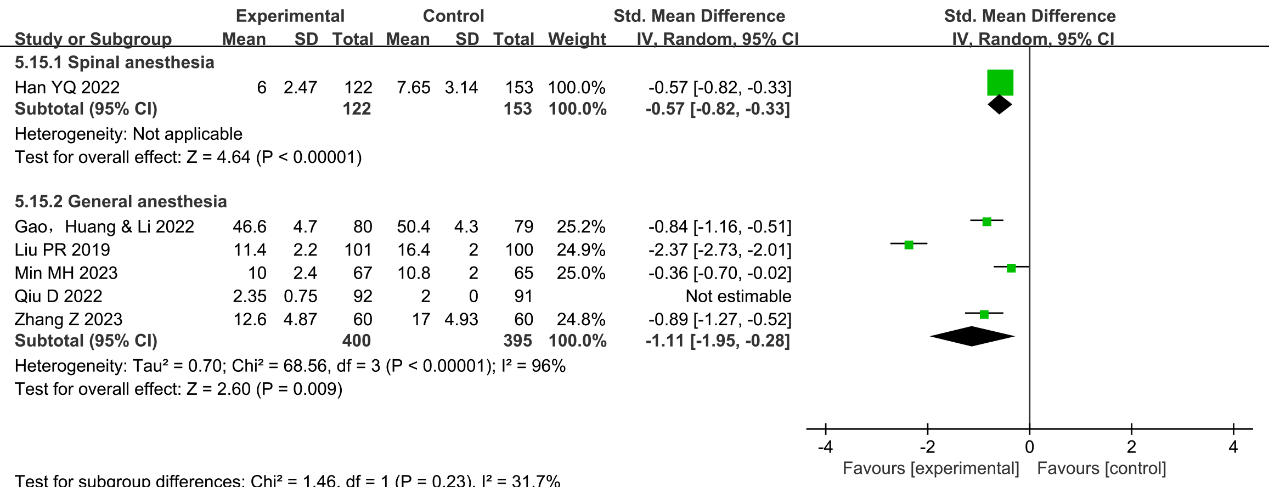
**

**Supplementary Figure 8. Forest plots of subgroup analyses.** Subgroup analysis for the scores of postoperative depression according to the presence or absence of preoperative depression (with preoperative depression vs. no preoperative depression) over the long term. **CI**, confidence interval. **df**, degrees of freedom.

**
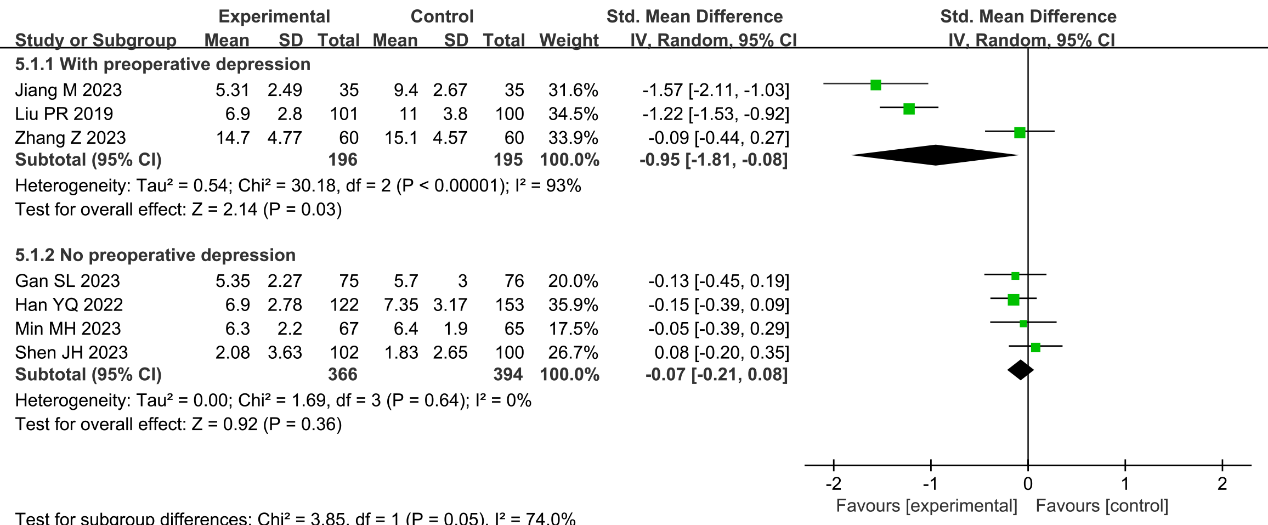
**

**Supplementary Figure 9. Forest plots of subgroup analyses.** Subgroup analysis for the scores of postoperative depression according to the type of anesthesia (spinal anesthesia vs. general anesthesia) over the long term. **CI**, confidence interval. **df**, degrees of freedom.

**
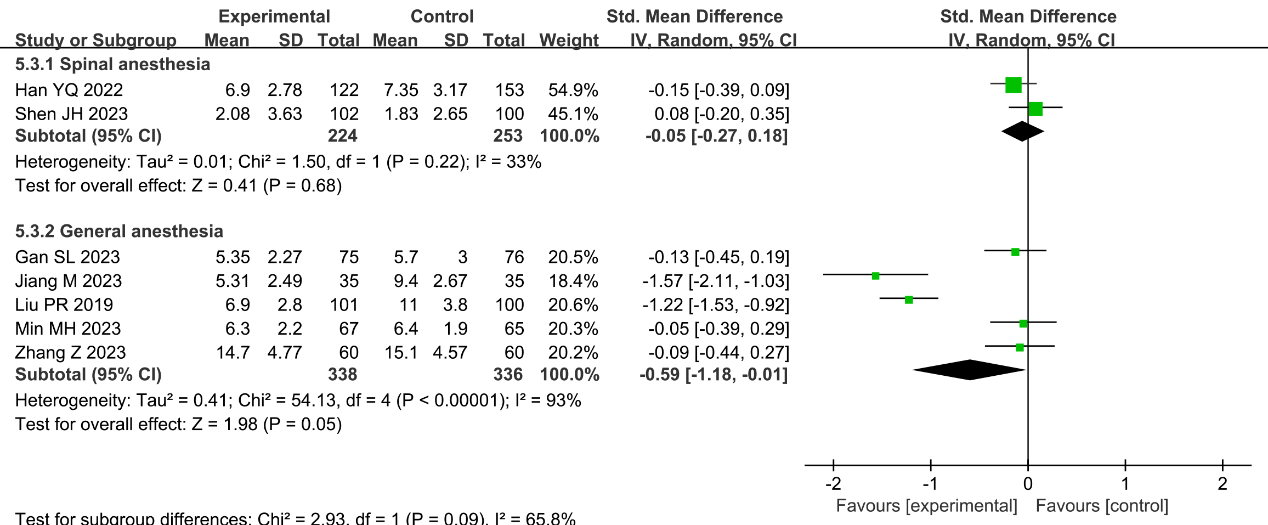
**

**Supplementary Figure 10. Forest plots of subgroup analyses.** Subgroup analysis for the scores of postoperative depression according to the dosage of esketamine administration (low dose vs. high dose) over the long term. **CI**, confidence interval. **df**, degrees of freedom.

**
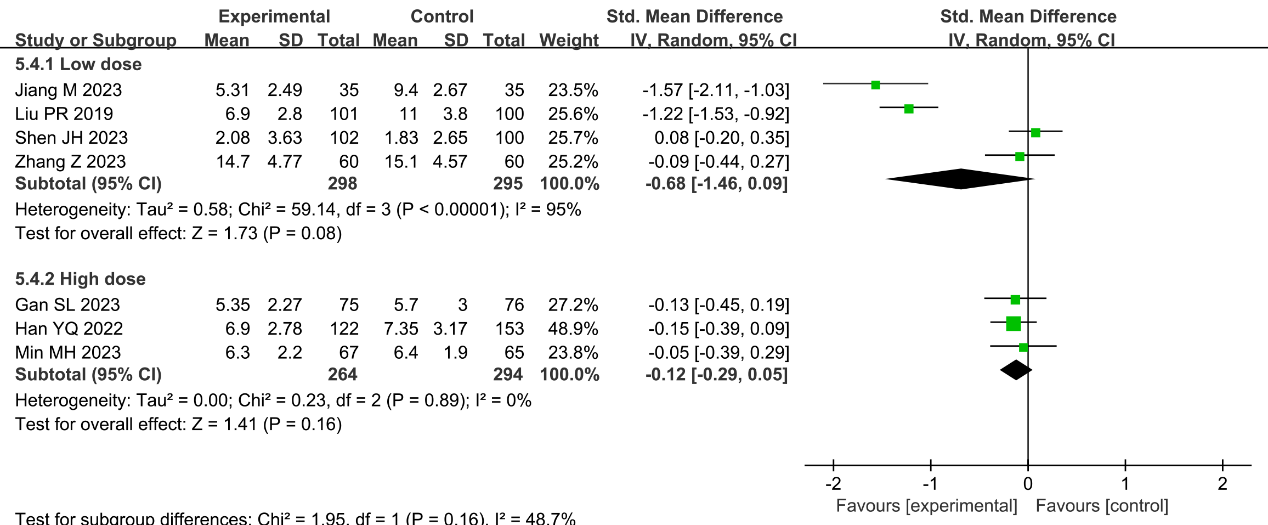
**

**Supplementary Figure 11. Forest plots of subgroup analyses.** Subgroup analysis for the scores of postoperative depression according to the method of esketamine administration (single administration vs. continue administration) over the long term. **CI**, confidence interval. **df**, degrees of freedom.

**
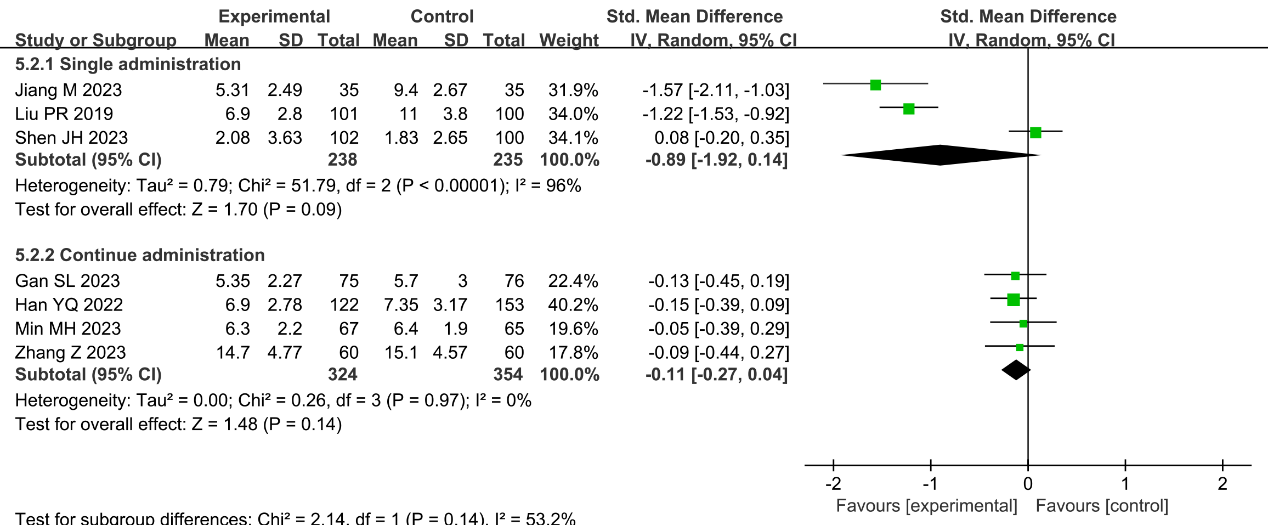
**

**Supplementary Figure 12. Leave-one-out sensitivity analysis.** Sensitivity analysis for the outcome of postoperative depression scores. A On the POD 1. **B** On the POD 3. **C** On the POD 7. **D** Over the long term. **CI**, confidence interval.


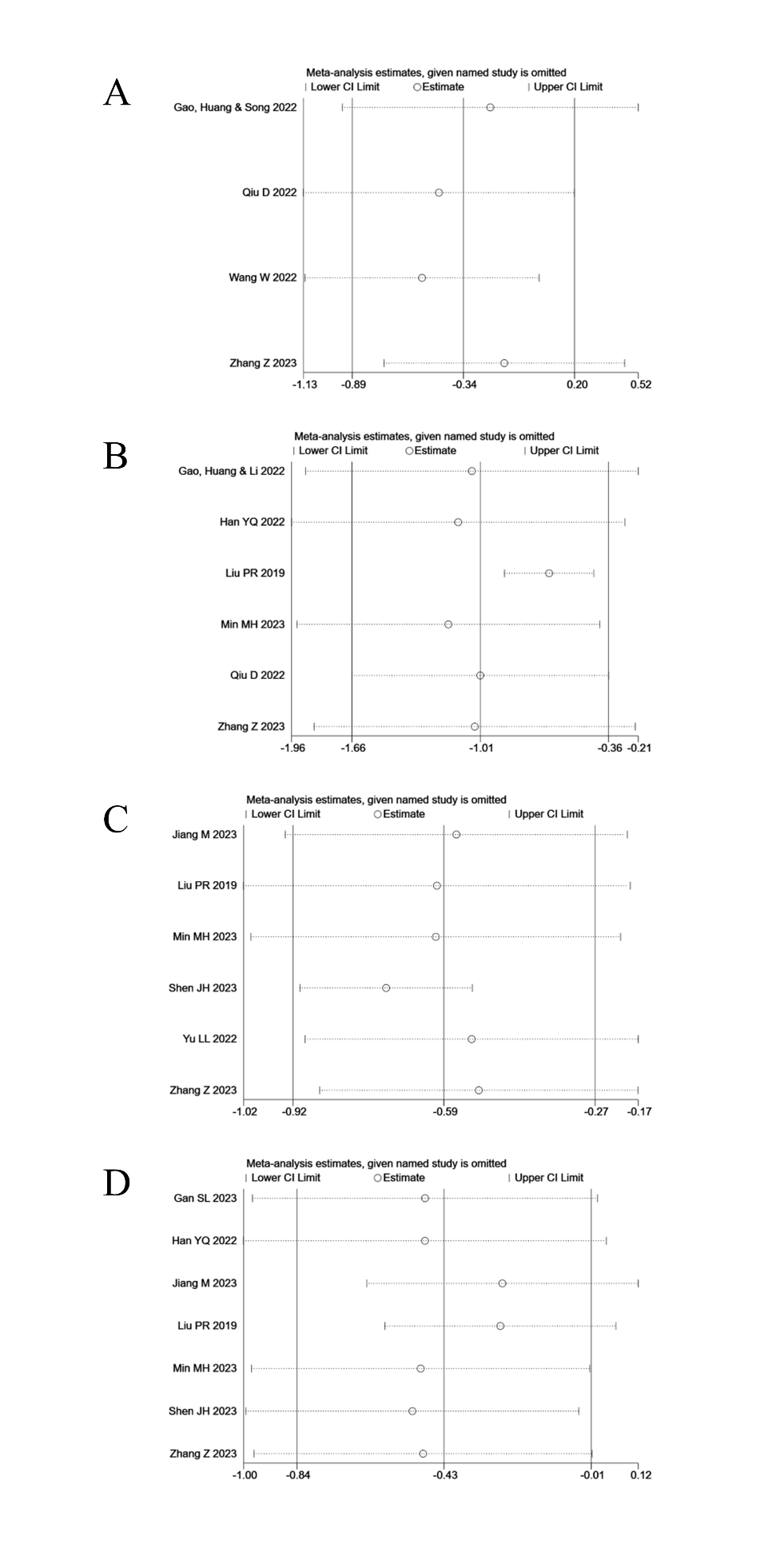


**Supplementary Figure 13. Leave-one-out sensitivity analysis.** Sensitivity analysis for the outcome of postoperative adverse effects. **A** Postoperative nausea and vomiting. **B** Dizziness. **C** Drowsiness. **D** Nightmare. **E** Dissociation. **CI**, confidence interval.


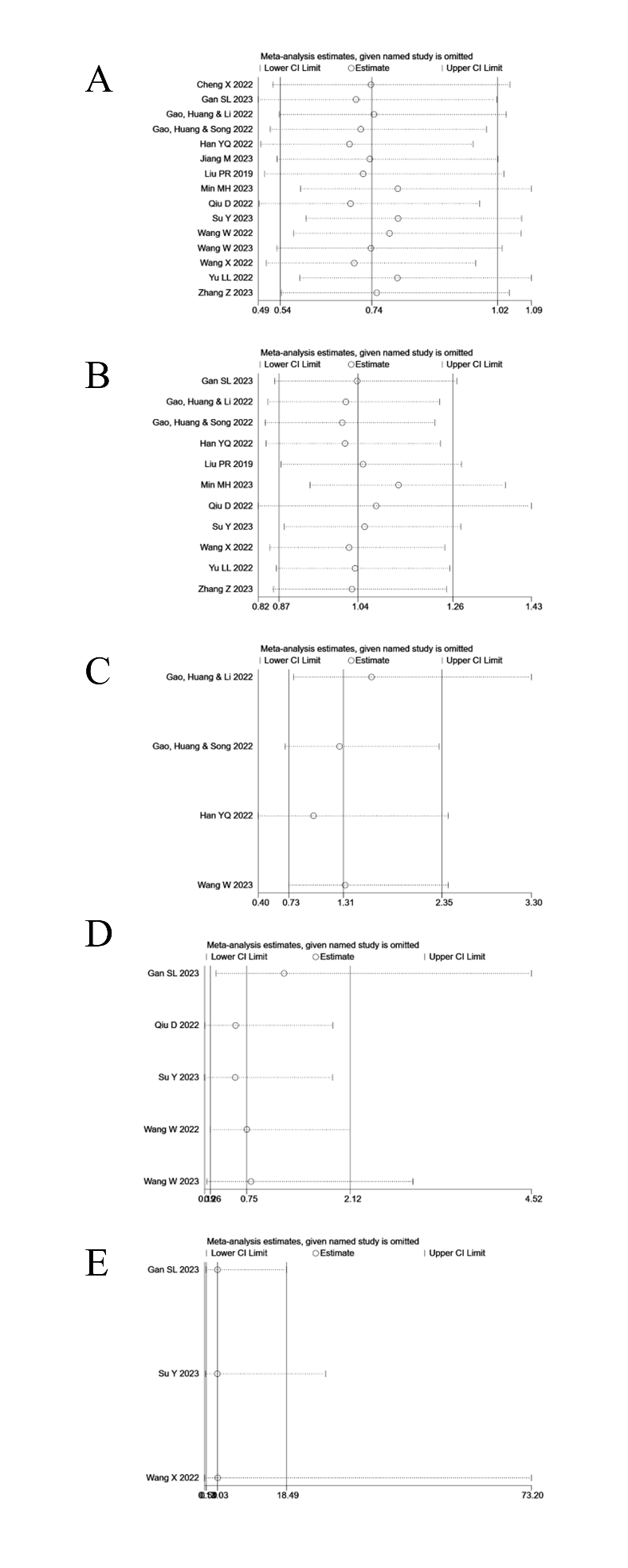


**Supplementary Figure 14. Leave-one-out sensitivity analysis.** Sensitivity analysis for the outcome of postoperative pain scores. A On the POD 1. **B** On the POD 2. **C** On the POD 7. **CI**, confidence interval.


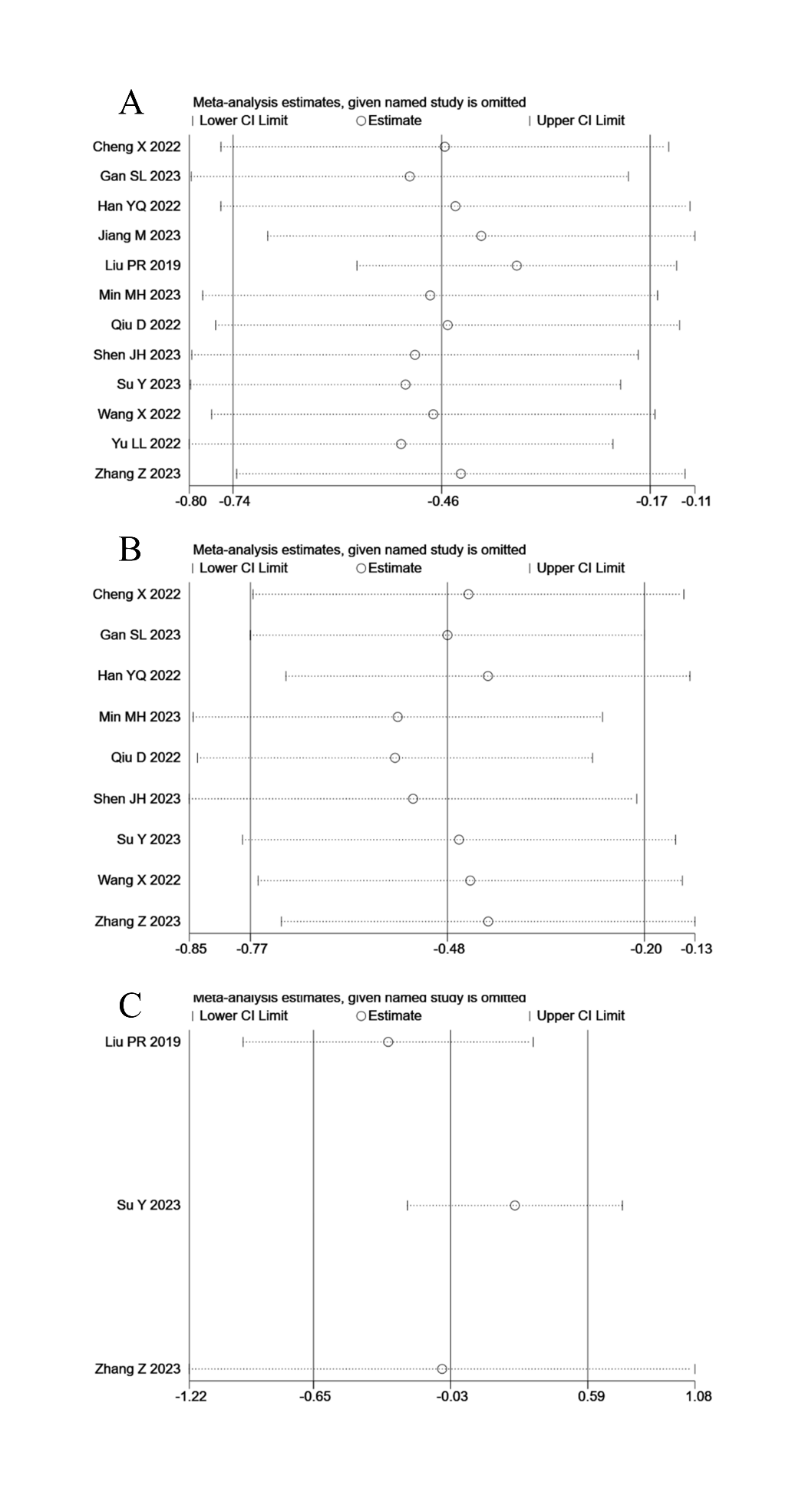

Supplement: Supplementary file 4 [file js9-111-1191-s004.docx]
