# Supplementary material for: Efficacy and safety of perioperative application of esketamine on postoperative depression: a meta-analysis of randomized controlled studies
Source: Int J Surg. 2024 Jun 27;111(1):1191–202. doi: 10.1097/JS9.0000000000001870 (PMC11745698; doi:10.1097/JS9.0000000000001870)
Supplement: Supplementary file 5 [file js9-111-1191-s005.docx]

**Supplementary Table 1: Summary of findings for the main comparison.**

| Esketamine for perinatal depression | | | | |
| --- | --- | --- | --- | --- |
| **Patient or population:** adults having surgery  **Setting:** hospital  **Intervention:** perioperative esketamine | | | | |
| **Comparison: esketamine vs. comparison** | | | | |
| **Outcome** | **Effect** | **Number of participants (number of studies)** | **Quality of evidence (GRADE)** * | **Comments** |
| Postoperative depression scores 1 day after surgery (SMD, 95% CI) | Two studies found that postoperative depression scores were lower in the esketamine group. | 521 (4 studies) | ⊕⊝⊝⊝ **very low** | Downgraded 3 levels because of very serious risk of bias, and serious inconsistency arising from heterogeneity. |
| Postoperative depression scores 3 days after surgery (SMD, 95% CI) | Six studies found lower postoperative depression scores in the esketamine group. | 1070 (6 studies) | ⊕⊕⊝⊝ **low** | Downgraded 2 levels because of very serious risk of bias. |
| Postoperative depression score 7 days after surgery (SMD, 95% CI) | Five of six studies found lower postoperative depression scores in the esketamine group. One study showed higher postoperative depression scores in the esketamine group. | 861 (6 studies) | ⊕⊕⊝⊝ **low** | Downgraded 2 levels because of serious risk of bias and serious inconsistency arising from heterogeneity between studies. |
| Postoperative depression score over the long term after surgery (SMD, 95% CI) | Six studies found lower postoperative depression scores in the esketamine group. One study showed a higher postoperative depression scores in the esketamine group. | 1151 (7 studies) | ⊕⊕⊝⊝ **low** | Downgraded 3 levels because of very serious risk of bias and serious inconsistency arising from heterogeneity between studies. |
| Postoperative nausea and vomiting (PONV) (RR, 95% CI) | Nine studies found a reduced risk of PONV with esketamine. Four studies showed an increased risk of PONV with esketamine. Two other studies found risk ratios of PONV close to favoring neither esketamine nor comparison. | 1989 (15 studies) | ⊕⊝⊝⊝ **very low** | Downgraded 4 levels because of very serious risk of bias, serious imprecision of results, and serious inconsistency arising from heterogeneity in PONV estimates. |
| Postoperative dizziness (RR, 95% CI) | Four study found a reduced risk of dizziness with esketamine. Seven other studies showed an increased risk of dizziness with esketamine. | 1649 (11 studies) | ⊕⊝⊝⊝ **very low** | Downgraded 4 levels because of very serious risk of bias, serious imprecision of results, and serious inconsistency arising from heterogeneity in dizziness estimates. |
| Postoperative drowsiness (RR, 95% CI) | Two studies found a reduced risk of drowsiness with esketamine. Two study showed an increased risk of drowsiness with esketamine. | 709 (4 studies) | ⊕⊝⊝⊝ **very low** | Downgraded 4 levels because of very serious risk of bias, serious imprecision of results, and serious inconsistency arising from heterogeneity in dizziness estimates. |
| Postoperative nightmare (RR, 95% CI) | Two studies found a reduced risk of nightmare with esketamine. Two study showed an increased risk of nightmare with esketamine. Two other studies found risk ratios of nightmare close to favoring neither esketamine nor comparison. | 599 (5 studies) | ⊕⊝⊝⊝ **very low** | Downgraded 3 levels because of serious risk of bias, serious imprecision of results, and serious inconsistency arising from heterogeneity in nightmare estimates. |
| Postoperative dissociation (RR, 95% CI) | Two studies found an increased risk of dissociation with esketamine. One study found risk ratios of dissociation close to favoring neither esketamine nor comparison. | 281 (3 studies) | ⊕⊝⊝⊝ **very low** | Downgraded 3 levels because of serious risk of bias, and very serious imprecision of results. |
| Postoperative pain (VAS or NRS) 24 hours after surgery (SMD, 95% CI) | Nine studies found lower postoperative pain scores in the esketamine group. Two studies showed higher postoperative pain scores in the esketamine group. One other study found postoperative pain scores close to favoring neither esketamine nor comparison. | 1677 (12 studies) | ⊕⊝⊝⊝ **very low** | Downgraded 3 levels because of very serious risk of bias, and serious inconsistency arising from heterogeneity in pain estimates. |
| Postoperative pain (VAS or NRS) 48 hours after surgery (SMD, 95% CI) | Six studies found lower postoperative pain scores in the esketamine group. One study showed higher postoperative pain scores in the esketamine group. Two other studies found postoperative pain scores close to favoring neither esketamine nor comparison. | 1268 (9 studies) | ⊕⊝⊝⊝ **very low** | Downgraded 3 levels because of very serious risk of bias, and serious inconsistency arising from heterogeneity in pain estimates. |
| Postoperative pain (VAS or NRS) 7 days after surgery (SMD, 95% CI) | One study found lower postoperative pain scores in the esketamine group. One study showed higher postoperative pain scores in the esketamine group. One other study found postoperative pain scores close to favoring neither esketamine nor comparison. | 388 (3 studies) | ⊕⊝⊝⊝ **very low** | Downgraded 3 levels because of serious risk of bias, serious imprecision of results, and serious inconsistency arising from heterogeneity in postoperative pain estimates. |
| **SMD**: standardized mean difference; **CI**: confidence interval;  **RR:** risk ratio; **NR**: no data reported | | |  |  |
| *GRADE Working Group grades of evidence  **High quality:** Further research is very unlikely to change our confidence in the estimate of effect  **Moderate quality:** Further research is likely to have an important impact on our confidence in the estimate of effect and may change  the estimate  **Low quality:** Further research is very likely to have an important impact on our confidence in the estimate of effect and is likely to  change the estimate  **Very low quality:** We are very uncertain about the estimate | | | | |
